# Bright light therapy in pregnant women with major depressive disorder: study protocol for a randomized, double-blind, controlled clinical trial

**DOI:** 10.1186/s12888-016-1092-2

**Published:** 2016-11-08

**Authors:** Babette Bais, Astrid M. Kamperman, Marjolein D. van der Zwaag, Gwen C. Dieleman, Hanneke W. Harmsen van der Vliet-Torij, Hilmar H. Bijma, Ritsaert Lieverse, Witte J. G. Hoogendijk, Mijke P. Lambregtse-van den Berg

**Affiliations:** 1 Department of Psychiatry, Erasmus University Medical Centre Rotterdam, ‘s Gravendijkwal 230, 3015CE Rotterdam, The Netherlands; 2 Epidemiological and Social Psychiatric Research Institute, Erasmus University Medical Centre Rotterdam, ‘s Gravendijkwal 230, 3015 CE Rotterdam, The Netherlands; 3 Department of Child and Adolescent Psychiatry, Erasmus University Medical Centre Rotterdam, ‘s Gravendijkwal 230, 3015 CE Rotterdam, The Netherlands; 4 Department of Obstetrics and Gynaecology, Erasmus University Medical Centre Rotterdam, ‘s Gravendijkwal 230, 3015 CE Rotterdam, The Netherlands; 5 Philips Lighting Research, High Tech Campus 7, 5656 AE Eindhoven, The Netherlands; 6 Research Centre Innovations in Care, Rotterdam University of Applied Sciences, Rochussenstraat 198, 3015 EK Rotterdam, The Netherlands; 7 Department of Psychiatry and Psychology, P. Debyelaan 25, 6229 HX Maastricht, The Netherlands

**Keywords:** Light therapy, Phototherapy, Depression, Depressive disorder, Pregnancy, Clinical trial, Circadian rhythm, Therapeutics

## Abstract

**Background:**

Depression during pregnancy is a common and high impact disease. Generally, 5–10 % of pregnant women suffer from depression. Children who have been exposed to maternal depression during pregnancy have a higher risk of adverse birth outcomes and more often show cognitive, emotional and behavioural problems. Therefore, early detection and treatment of antepartum depression is necessary. Both psychotherapy and antidepressant medication, first choice treatments in a non-pregnant population, have limitations in treating depression during pregnancy. Therefore, it is urgent and relevant to investigate alternative treatments for antepartum depression. Bright light therapy (BLT) is a promising treatment for pregnant women with depressive disorder, for it combines direct availability, sufficient efficacy, low costs and high safety, taking the safety for the unborn child into account as well.

**Methods:**

In this study, 150 pregnant women (12–18 weeks pregnant) with a DSM-V diagnosis of depressive disorder will be randomly allocated in a 1:1 ratio to one of the two treatment arms: treatment with BLT (9.000 lux) or treatment with dim red light therapy (100 lux). Both groups will be treated for 6 weeks at home on a daily basis for 30 min, within 30 min of habitual wake-up time. Follow-up will take place after 6 weeks of therapy, 3 and 10 weeks after end of therapy, at birth and 2, 6 and 18 months postpartum. Primary outcome will be the average change in depressive symptoms between the two groups, as measured by the Structured Interview Guide for the Hamilton Depression Scale – Seasonal Affective Disorder version and the Edinburg Postnatal Depression Scale. Changes in rating scale scores of these questionnaires over time will be analysed using generalized linear mixed models. Secondary outcomes will be the changes in maternal cortisol and melatonin levels, in maternal sleep quality and gestational age, birth weight, infant behaviour, infant cortisol exposure and infant cortisol stress response.

**Discussion:**

If BLT reduces depressive symptoms in pregnant women, it will provide a safe, cheap, non-pharmacological and efficacious alternative treatment for psychotherapy and antidepressant medication in treating antepartum depression, without any expected adverse reactions for the unborn child.

**Trial registration:**

Netherlands Trial Register NTR5476. Registered 5 November 2015.

## Background

Depression during pregnancy is a common and high impact disease. Approximately 5–10 % of pregnant women suffers from depression [1], which has been confirmed by a study in Rotterdam, the second largest city in the Netherlands [2]. Children who are exposed to maternal depression during pregnancy have a higher risk of adverse birth outcomes, such as low birth weight, and more often show cognitive, emotional and behavioural problems [3–6]. The perinatal period is a critical period, in which epigenetic programming determines not only the perinatal health, but also that of following generations [7]. Therefore, early detection and prompt treatment of depression during pregnancy can benefit both mother and child.

In non-pregnant women, guidelines propose psychotherapy, antidepressant medication or a combination of both as treatment for depression. However, clinical practice shows limited relevance of these guidelines during pregnancy, as the direct availability of psychotherapists is poor, postponing treatment for several months or more. In pregnancy, the window of opportunity is small and from the perspective of the child postponement is in fact non-treatment. Other limitations of psychotherapy are its dependence on good language skills, absence of problems that limit access to therapy and a strong motivation to reflect on ones emotions, cognition and behaviour. These factors limit the applicability of psychotherapy in a majority of pregnant women with depression, who share a socioeconomic deprived background and often have coexisting problems interfering with compliance [8]. Therefore, women with depression during pregnancy may be treated with antidepressants. In North America, use of antidepressants during pregnancy is reported by 5–13 % of pregnant women [9, 10]. In the Netherlands, 2–3 % of pregnant women use antidepressant medication [11, 12]. However, the safety of these medications during pregnancy is controversial [13, 14].

Therefore, investigating non-pharmacological approaches to treating depression during pregnancy is urgent and relevant, for both mother and child. Bright light therapy (BLT) is a promising treatment for pregnant women with depression based on several theoretical and clinical considerations, which will be discussed below.

### BLT and depression

BLT is the first choice treatment for seasonal affective disorder (SAD) [15], a condition of reoccurring depressions during fall and winter, with remissions in spring and summer [16].

The effects of BLT have not only been consistently shown on SAD [16–19], but also on other diseases, such as non-seasonal depression [17], adult attention-deficit/hyperactivity disorder [20] and bulimia nervosa [21, 22]. The effects of BLT on non-seasonal depression have been shown in various populations, like elderly residents of group care facilities and patients with Alzheimer’s disease [23–25].

BLT has been shown to synchronize the biological clock with the environmental day-night rhythm and to shift the circadian rhythm [15, 16]. Evidence suggests that this mediates the effects of BLT on depression, which has been indirectly supported by enhanced sleep and rhythms of melatonin and cortisol [23].

### Hypothalamus-pituitary-adrenal axis

**Fig. 1:**
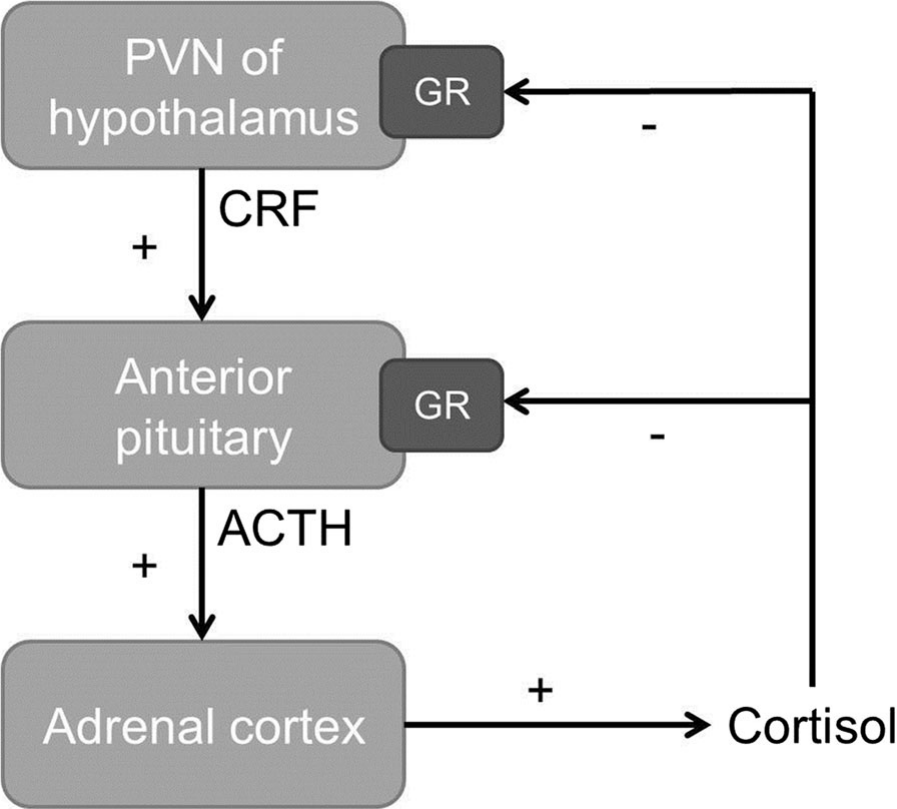
Schematic diagram of the hypothalamus-pituitary-adrenal gland (HPA) axis. Shown are the different structures and hormones involved in the HPA-axis. CRF, produced and released by the hypothalamus, stimulates the anterior pituitary to produce and release ACTH, which in turn stimulates the production and release of cortisol by the adrenal cortex. Cortisol inhibits both the hypothalamus and pituitary through the GR. PVN = paraventricular nucleus; GR = glucocorticoid receptor; CRF = adrenocorticotrophic hormone releasing factor; ACTH = adrenocorticotrophic hormone; + = stimulating; − = inhibiting

The hypothalamus-pituitary-adrenal gland (HPA) axis is involved in the synchronization of the biological clock by BLT. This axis regulates the secretion of cortisol in response to stress [26]. HPA-axis activity is controlled by the corticotropin-releasing factor (CRF) secreted by parvocellular neurosecretory cells in the paraventricular nucleus (PVN) of the hypothalamus, which activates the secretion of adrenocorticotrophic hormone (ACTH) from the anterior pituitary, which in turn stimulates the production and release of cortisol from the adrenal cortex [26, 27]. CRF and ACTH are both inhibited by cortisol through the glucocorticoid receptor (GR) [26, 27]. Figure 1 shows a schematic overview of this feedback loop.

The cause of depression is largely unknown. However, the HPA-axis is thought to play a crucial role in the pathophysiology – as cause or consequence, since hyperactivity of this axis is associated with depression. More specifically, depression is thought to be related to reduced inhibition by cortisol, due to impaired GR function [27]. This is supported by a post-mortem study among depressed patients, which showed hyperactivity of CRF neurons of the hypothalamic PVN [28]. Second, increased basal cortisol levels are commonly found in patients with depression [27].

### Suprachiasmatic nucleus

**Fig. 2:**
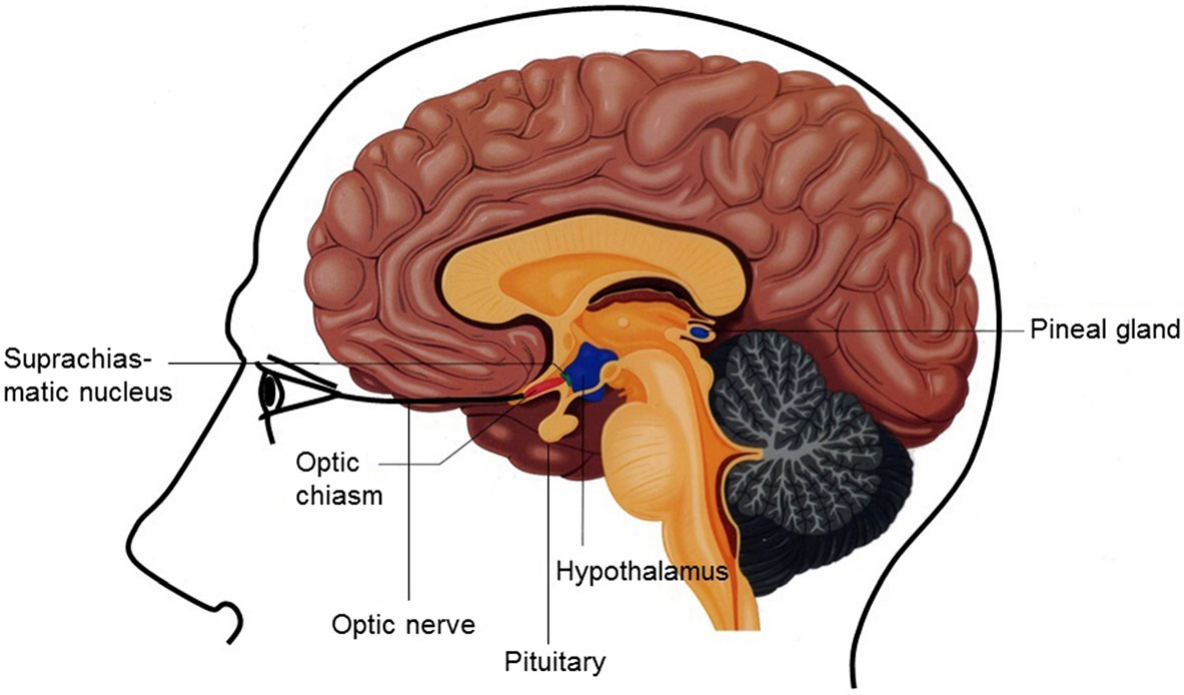
Sagittal view of the brain. This figure shows a sagittal view of the suprachiasmatic nucleus, the optic chiasm, the optic nerve, the hypothalamus, the pituitary and the pineal gland

The suprachiasmatic nucleus (SCN), known as the ‘biological clock’, controls the HPA-axis: decreased inhibitory control of the SCN on the HPA-axis has been shown to be associated with HPA-axis hyperactivity [29]. The SCN is located in the hypothalamus on top of the optic chiasm and is the central pacemaker of all physiological and behavioural rhythms [15, 30]. Light is the most powerful environmental signal that synchronizes the SCN with the environmental day-night rhythm (also known as ‘zeitgeber’). Environmental light versus darkness is signalled to the SCN by melanopsin-containing retinal ganglion cells through the retino-hypothalamic tract in the optic nerve (Fig. 2) [15, 31]. The SCN is able to maintain circadian rhythms, even in the absence of zeitgebers [15, 30]. This central control function of circadian rhythms is lost when the SCN is damaged or obliterated. Mice studies have shown that ablation of the SCN results in arrhythmicity [32, 33]. A case report of a patient with hypothalamic damage demonstrated disturbances in the sleep-wake cycle, body temperature and cognitive and behavioural functioning [34].

### Melatonin

Melatonin is, next to cortisol, influenced by light. Melatonin is produced and secreted by the pineal gland and, like other circadian rhythms, its rhythm is controlled by the SCN (Fig. 2) [35, 36]. Typically, melatonin levels rise in the evening, peak at early morning hours and drop to baseline at awakening [26]. Light inhibits the production of melatonin [26].

Different studies showed a change in melatonin secretion in psychiatric diseases, such as a reduction of melatonin secretion in depression [35, 37]. Earlier, BLT in the morning has been shown to normalize saliva melatonin evening levels in elderly patients with a depressive disorder [23].

Melatonin concentrations increase during the course of pregnancy [36]. However, a study showed that nocturnal melatonin levels were lower in depressed pregnant women, compared to healthy controls [38]. In this study, we want to explore the effects of BLT on evening and morning melatonin levels.

### BLT and depression during pregnancy

The SCN generates the circadian rhythms in physiology and behaviour, including reproductive hormones. Pregnant women typically show disturbed, desynchronised circadian rhythms, resulting in disturbed sleep patterns, which puts them at risk for depression [39]. Moreover, disturbed sleep and decreased physical condition put pregnant women at risk for decreased activity and less exposure to daylight [40], which might further enhance their risk for depression.

Two small (*n* = 10 and *n* = 27) randomized controlled trials among pregnant women with non-seasonal depression showed significant improvement of depression among women exposed to BLT compared to placebo [41, 42]. Treatment effect in terms of mean improvement of depressive symptoms was comparable with the effects of antidepressant medication (effect size around 0.45), making it a competitive treatment for antepartum depression, but without the possible adverse effects of medication to the unborn child. Although these studies provide evidence for the effectiveness of BLT for depression during pregnancy, their sample size is small.

While in previous studies among elderly patients cortisol and melatonin rhythms normalized after BLT treatment [23], the question is whether improvement of depressive symptoms after BLT in pregnant women is also mediated through improved endocrine functioning or whether these symptoms are primarily determined by the physiology of pregnancy itself. Therefore, we will also examine the circadian rhythms and hormone levels of the pregnant women, to study whether BLT also effects endocrine functioning during pregnancy.

From previous research, we know that maternal depression during pregnancy negatively influences intra-uterine and postnatal child development [3–6, 43–47]. How intra-uterine child development is influenced by maternal depression has yet to be determined. Possible mechanisms are maternal cortisol crossing the placenta, placental secretion of CRH – which has a positive feedback loop with maternal and foetal cortisol – and reduced blood flow to the foetus, causing foetal growth restriction [5, 43–45, 47]. Increased maternal levels of cortisol, as a cause and/or consequence of maternal depression during pregnancy, might program the intra-uterine developing HPA-axis of the child, making it susceptible to increased stress reactivity in future life [3, 4, 48], which has also been confirmed in animal studies [49]. Therefore, where the earlier conducted studies on antepartum depression and BLT only studied the effects of BLT on mood [41, 42], it will be interesting to examine the effects on infant stress reactivity, long-term cortisol exposure and infant behaviour.

### Aims

In this study, we will primarily study the effects of BLT on depression during pregnancy, including adverse effects. Second, we will study whether this clinical improvement is accompanied by improved sleep quality and normalized melatonin and cortisol levels during pregnancy. Third, we will study the effects of BLT on gestational age, birth weight, infant behaviour, infant cortisol stress response and long-term cortisol exposure of the infant.

## Methods/Design

### Hypotheses

Primary hypothesis: Daily treatment with 6 weeks of morning BLT improves depressive symptoms during pregnancy.

Secondary hypotheses:

1. Clinical improvement of depressive symptoms is accompanied by improved sleep patterns, lower basal cortisol levels and normalized melatonin concentrations during pregnancy.
2. Treatment with BLT during pregnancy improves birth and child outcomes: higher gestational age, higher birth weight, less regulation problems in infants and lower cortisol stress response. In addition, infants will show lower long-term cortisol exposure.

### Study overview

This study is a randomized, double-blind, placebo-controlled clinical trial. After baseline measurements (T0), the participants will be randomly allocated to either receive active BLT or dim red light therapy (DRLT) in a 1:1 ratio. Subsequently, they will commence their daily treatment with light, which takes place at the participants’ home for 6 weeks. After treatment, follow-up will take place at the following time points:

- after 6 weeks of treatment, which marks the end of treatment (T1);
- 3 weeks after end of treatment (T2);
- 10 weeks after end of treatment (T3);
- at birth (T4);
- 2 months postpartum (T5);
- 6 months postpartum (T6);
- 18 months postpartum (T7).

**Fig. 3:**
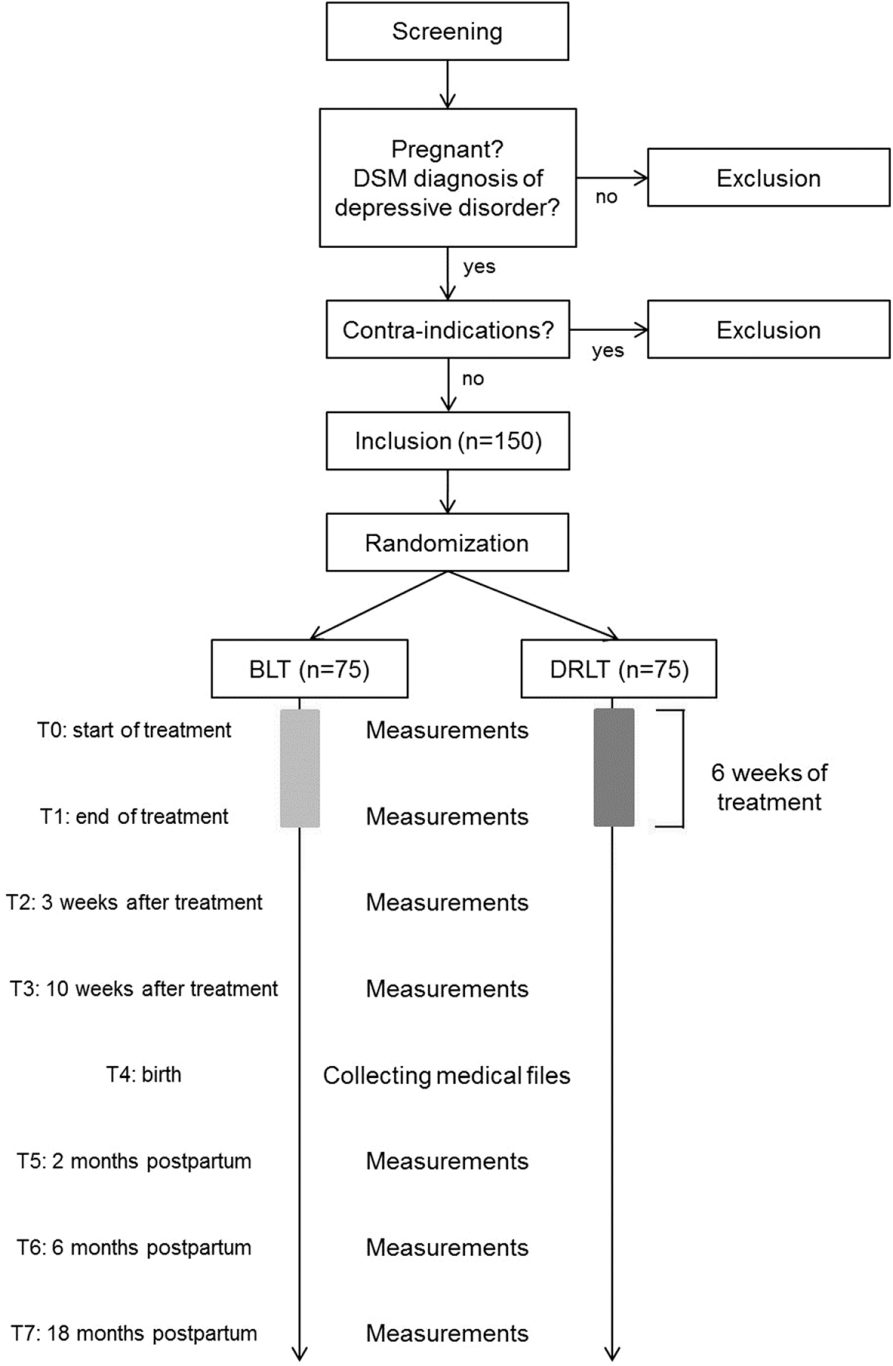
Flowchart of overview study. DSM = Diagnostic and Statistical Manual of Mental Disorders; BLT = bright light therapy; DRLT = dim red light therapy

At these time points, questionnaires, body material and information from medical files will be collected (Table 1). A flowchart of this study is shown in Fig. 3.

**Table 1:** Overview assessment of questionnaires and collection of body material and medical files per time point

	T0	IP ^a^	T1	T2	T3	T4	T5	T6	T7
Questionnaires									
SIGH-SAD	X	X	X	X	X		X	X	X
EPDS	X	X	X	X	X		X	X	X
Life events	X	X	X	X	X		X	X	X
PSQI	X	X	X	X	X		X		
User expectations	X								
User experiences			X				X		
MABS							X		
CBCL									X
Body material									
Urine cortisol	X		X	X			X		
Saliva cortisol/melatonin	X		X	X			X		
Hair cortisol	X				X		X		
Saliva cortisol (infant)							X		
Hair cortisol (infant)							X		
Actigraphy	X ^b^				X ^c^		X ^c^		
Collecting medical files						X			

*T0* baseline (start of treatment), *T1* after 6 weeks of treatment (end of treatment), *T2 = 3* weeks after treatment, *T3 = 10* weeks after treatment, *T4* birth, *T5* 2 months postpartum, *IP* intervention period, *SIGH-SAD* Structured Interview Guide for the Hamilton Depression Scale – Seasonal Affective Disorder version, *EPDS* Edinburgh Postnatal Depression Scale, *PSQI* Pittsburgh Sleep Quality Index, *MABS* Mother and Baby Scales, *CBCL* Child Behaviour Checklist

^a^In the intervention period, the questionnaires will be assessed weekly

^b^The actiwatch needs to be worn for 8 weeks at T0

^c^At T3 and T5, the actiwatch needs to be worn for 9 days

### Participants

In this study, pregnant women with a depressive disorder will be eligible for participation. The specific inclusion and exclusion criteria of the study are listed in Table 2.

**Table 2:** Inclusion and exclusion criteria

Inclusion criteria	Women
	18–45 years of age
	12–18 weeks pregnant
	DSM-V diagnosis of depressive disorder (as assessed by the Structured Clinical Interview for DSM disorders)
Exclusion criteria	Insufficient proficiency in Dutch or English
	Multiple pregnancy
	The use of antidepressants shorter than 2 months
	Bipolar I or II disorder
	Any psychotic episode
	Substance abuse
	Primary anxiety disorder
	Recent history of suicide attempt
	Shift-work
	Somatic and/or obstetric conditions that override study participation
	Previous treatment with BLT
	Eye condition (macular degeneration, eye diseases, recent eye surgery)

### Recruitment

In the Netherlands, maternity care for low-risk pregnancies is provided in primary care, which is midwife-led. High-risk pregnancies are cared for in a general hospital (secondary care) or foetal-maternal medicine unit (tertiary care).

In this study, women will be mainly recruited through both midwifery practices and hospitals participating in the South West Consortium in the Netherlands. This is a unique consortium in which almost all parties involved in perinatal care in the South West region of the Netherlands are united: midwives, obstetricians, paediatrician, and several public health institutes. The consortium covers both urban areas (such as the city Rotterdam) and rural areas. Previous studies in this consortium involved screening for psychosocial risk factors, psychiatric disease and the impact of structuring psychosocial care. Rotterdam is the second largest city in the Netherlands with more than 620.000 inhabitants [50]. It has a high number of deprived neighbourhoods, defined as 10 % of pregnant women having a low socio-economic status (<20^th^ percentile) [51].

Women will be routinely screened on psychopathology and psychosocial problems during their first prenatal visit in midwifery practices and hospitals by a screening model, the Mind2Care [2]. During this screening, women are asked to fill out a web-based questionnaire, consisting of 33 items, covering four domains: a socio-demographic, obstetric, psychiatric and psychosocial domain, including the Edinburgh Postnatal Depression Scale (EPDS) [52]. A cut-off score of 9 or above of the EPDS is used by the Mind2Care, in order to refer a woman towards tailored mental health care. Sensitivity and specificity of the EPDS are respectively 86 and 78 % [52].

In addition, women who visit our outpatient psychiatric clinic – a centre of excellence in perinatal psychiatry – at the Erasmus University Medical Centre in Rotterdam for their depressive symptoms will be offered to participate in our study when they are not fully remitted from depressive symptoms after 2 months of treatment with antidepressant medication and/or other psychiatric treatment.

Third, pregnant women who visit their general practitioner (GP) for depressive symptoms may be referred to the study by their GP.

Finally, women will be recruited via (social) media, such as press releases, so women with depressive symptoms can enrol in the study without referral from their midwife, gynaecologist, GP or mental health care worker.

### Ethics

This study will be conducted in accordance with the Helsinki Declaration, meaning that participation is voluntary and written informed consent will be obtained. Before entering the study, subjects will receive information about the study and its risks and benefits, verbal and in writing. Subjects will have a reflection period of one week. Participants can leave the study at any time for any reason without consequences with regard to their current or future treatment. Also, the investigator can decide to withdraw a participant from the study for urgent medical reasons.

The study has been approved by the medical ethical committee of the Erasmus University Medical Centre, Rotterdam, The Netherlands (registration number MEC-2015-731). A Data Safety Monitoring Board has been installed, which monitors the safety of this research.

In case of adverse effects, a treatment protocol will be effective.

### Randomization and blinding

We will randomly assign 150 participants in a 1:1 allocation ratio to either receive BLT or DRLT. Randomization will be done with the web-based computer-generated randomization schedule ALEA (software for randomization in clinical trials, version 2.2) using random block sizes of 2 to 6. Stratification factors will be the use of any antidepressant medication and the number of previous depressive episodes. This will be dichotomized to 3 or less previous depressive episodes versus 4 or more [53].

Participants will be blinded for their allocation. They will be informed that this study examines the efficacy of light therapy with two different colours.

Participants will be asked to guess to which treatment group they are allocated to after treatment, as suggested by the Cochrane’s Collaboration’s tool for assessing risk of bias in randomized trials [54].

Blinded, independent assessors will be involved in the outcome ratings and will conduct the interviews at T1, T2, T3, T5, T6 and T7 and on a weekly base during the intervention period. The participants are asked not to share any details about their treatment towards the assessors. In case information is shared, the assessor will be replaced. After each interview, the assessors will be asked to guess the allocation of the participant.

The researcher who will perform the primary statistical analysis (AK) will be blinded for allocation.

The field researcher (BB) will install the lamps and will provide the participants with instructions. Also, the field researcher will answer any questions asked by participants. For this, we will use protocolled answers, to maintain the blindness of the participants. Moreover, the field researcher will ask the participants about side-effects at T1 and on a weekly base during the intervention, keeping the independent assessors blinded for adverse effects that might break the blinding, e.g. strained eyes. For these practical reasons, the field researcher will not be blinded.

### Intervention

Participants will be randomly allocated to BLT (9.000 lux) or DRLT (100 lux). Treatment will take place daily at home for 6 weeks, starting at 12–18 weeks pregnancy. Participants will be asked to commence the treatment within 30 min of habitual wake-up time with a duration of 30 min. Participants will sit in front of two light boxes with a distance of approximately 40 cm. The light boxes will be placed in a custom-made scaffolding. In this way, the height of the light boxes can be adjusted per person, ensuring the same distance. Also, the scaffolding ensures lighting from above, which avoids glare [42]. This makes the treatment more comfortable, enhancing treatment adherence.

The active dose was found effective in other studies [23, 41, 42]. Dim red light can be considered to be biologically inactive [55]. Although a Cochrane review of studies in BLT in non-seasonal depression showed that BLT may be effective in as little as 1 week [55], we will choose 6 weeks of daily light exposure, since the 2 studies among pregnant women with non-seasonal depression showed significant effects of BLT from 5 weeks treatment [41, 42].

All participants in both treatment arms will receive treatment as usual. Women are always free to visit their GP, whenever they are in need of this. The GP is always free to start treatment if he/she feels necessary.

When depressive symptoms increase and/or in the case of suicidal ideation, an action plan is set up and appropriate measures will be taken.

Different measures have been taken to enhance treatment adherence:

- A sensor that measures the amount of lux perceived is installed in the actiwatches, which will monitor the therapy adherence.
- Since the lamps will be installed into a custom-made scaffolding that ensures lighting from above, treatment will be more comfortable, which enhances treatment adherence.

### Outcome measures

The primary outcome measure will be the average change in depressive symptoms between the two groups, as measured by the Structured Interview Guide for the Hamilton Depression Scale – Seasonal Affective Disorder version (SIGH-SAD) and the EPDS at different time points. Second, we will study responders vs. non-responders, where response is defined as a ≥50 % decrease to a final score of ≤ 8 on the 17-item Hamilton scale and ≤ 5 on the EPDS.

The secondary outcome measures of this study will be the changes in maternal cortisol and melatonin levels (1), in maternal circadian rhythm (2) and in birth and child outcomes (3).

1. Morning and evening cortisol levels in saliva will be measured as a measure for HPA-axis activity. Morning and evening melatonin levels in saliva will be measured as measure of a participant’s circadian phase position. Outcome measures will be the changes in cortisol and melatonin saliva levels between the two groups.
  Total 24-h cortisol excretion will be determined from urine. Outcome measure will be the changes in cortisol levels within and between the two groups.
2. Hair will be used as a long-term measure for cortisol excretion. Changes in levels between the two groups will be used as outcome measure.
3. Through actiwatches and a structured questionnaire (the Pittsburgh Sleep Quality Index – PSQI), information will be obtained regarding total sleep time, sleep efficiency and sleep onset latency, as well as circadian estimates from the rest activity rhythm such as intradaily and interdaily variability and rhythm amplitude.
4. Differences in birth and child outcomes between the two groups will be used as outcome measures: pregnancy duration, birth weight, child behaviour, long-term cortisol exposure and cortisol stress response at a routine vaccination.

Finally, we will ask about user expectations and user experiences.

Complete follow-up will be pursued. In case of discontinuing or deviating from the intervention, we will collect outcomes of EPDS and SIGH-SAD assessment.

### Sample size

Based on previous literature [23, 41, 56], we expect a small to moderate response (time x treatment interaction on depressive symptoms). This corresponds to a 10 to 15 % reduction of depressive symptoms over the full course of treatment. To demonstrate this (with an α of 0.05 and a β of 0.80), we will need a sample size of 63 participants per arm (126 in total). To account for lost to follow up during and after treatment, we will aim at including 150 participants. Power calculations were performed using GLIMMPSE 2.1.5 software [57].

In case of withdrawal of a participant during the recruitment period, another participant will be recruited to obtain the aimed number of participants.

### Adverse effects

At every measurement, we will ask for adverse effects. The adverse effects of BLT, such as headache and nausea, are generally mild and short-lived [58, 59]. A switch to hypomania is a more serious adverse effect, which would require effect managing. In a study exploring the side effects of short-term 10,000 lux light therapy in 70 patients suffering from SAD, 1 subject experienced hypomania [58]. In the two studies (*n* = 10 and *n* = 27) studying the effects of light therapy in a pregnant population, 1 subject showed hypomanic symptoms [41, 42]. If a participant shows hypomanic symptoms, the daily treatment duration will be reduced. This enhances the clinical safety [41].

No adverse effects for the foetus will be expected [42, 56].

### Inclusion

Women will be asked to provide the following baseline socio-demographic factors: age, ethnicity, level of education, marital status, parity, unplanned pregnancy, body mass index, somatic conditions (if not exclusion), medication use and substance use (smoking, alcohol, drugs).

The GP will be contacted to verify whether the participant meets any exclusion criteria. The results will be discussed with an experienced perinatal psychiatrist (ML), who will – as a safety measure – verify the diagnosis and inclusion and exclusion criteria. If there are no clinical contraindications, randomization will take place.

After a positive screening on the EPDS, eligible women will be interviewed to assess lifetime psychiatric diagnosis. This will be done with the Structured Clinical Interview for DSM disorders (SCID), a semi-structured interview that is considered to be the golden standard for making the major DSM-V axis I psychiatric diagnoses [60].

### Measurements

#### Primary outcome measures

Depressive symptoms during pregnancy will be assessed using the SIGH-SAD and the EPDS.

### SIGH-SAD

The SIGH-SAD is a 29-item structured interview and consists of 21 HAM-D (Hamilton Rating Scale for Depression) items and 8 atypical items, of which 11 items can be scored with a value of 0–2, 5 items with a value of 0–3 and 13 items with a value of 0–4 [61]. The sum score ranges from 0 to 63 for the HAM-D items and from 0 to 26 for the atypical items, resulting in a total sum score of 0 to 89 [61].

We will choose the original 17-item HAM-D questionnaire as primary measure, since it is more commonly used in clinical practice and research. Interrater reliability for the 17-item HAM-D questionnaire ranges from 0.82 to 0.98 [62]. Sensitivity and specificity are respectively 0.76 and 0.91 [62]. Positive and negative predictive value are respectively 0.77 and 0.92 [62]. Next to the original 17-item HAM-D questionnaire, we will use the entire SIGH-SAD questionnaire, since this questionnaire is the current benchmark for assessment of severity of depression in light therapy trials.

### EPDS

The EPDS, a structured 10-item questionnaire, will be used as a validated self-report measure of depression during pregnancy [52, 63]. Each item is scored with a value, ranging from 0 to 3, which leads to a sum score of 0 to 30 [52]. Sensitivity and specificity of the EPDS are respectively 86 and 78 % [52]. Originally, the EPDS was developed for the detection of postnatal depression, but has been validated for screening depression during pregnancy as well [63]. In this study, we will use a cut-off of 9, in accordance with the screening tool, the Mind2Care [2].

#### Secondary outcomes (endocrine)

Endocrine levels during pregnancy will be studied measuring saliva melatonin and cortisol in urine, saliva and hair.

### Urinary free cortisol

Urinary free cortisol levels during a 24-h period provide a non-invasive valid estimation of overall daily cortisol production [64]. Urine will be collected starting after the first voided urine after awakening and will include the first voided urine on the following day. The cortisol level will be determined by radioimmunoassay. Completeness of collection will be ascertained by interviews documenting urine losses. Only complete collections, with creatinine within the normal range of 0.06 mg/dL per 24 h will be included in the analysis.

### Saliva cortisol

As a measure of HPA-axis activity, saliva cortisol will be collected using cotton dental rolls, including 4 sequential samples at 30-min intervals starting at awakening and 3 sequential samples at hourly intervals starting 2 h before predicted bedtime with the last sample at bedtime. The samples will be collected the following day and subsequently delivered to the laboratory, where they will be centrifuged and stored at −80 °C. Samples will be analysed using a cortisol assay on an immunoanalyser system. For determination of the diurnal time course of saliva cortisol levels, only days with at least 6 valid samples will be included in analyses.

### Hair cortisol

Hair cortisol will be assessed as a validated biomarker for long-term cortisol exposure. The maternal hair strands will be collected and processed according to existing methods [65]. With this method, cortisol levels can be retrospectively assessed depending on hair length (i.e. one month for each centimetre of hair). Norm data from healthy pregnant controls will be available through one of our other ongoing studies.

### Saliva melatonin

As a measure of a participant’s circadian phase position, saliva melatonin levels will be collected using cotton dental rolls at hourly intervals with 3 sequential samples, starting 2 h before predicted bedtime with the last sample at bedtime, under dim light conditions. One sample will be taken at awakening. The samples will be collected the following day and subsequently delivered to the laboratory, where they will be centrifuged and stored at −80 °C. All samples will be analysed using Liquid Chromatography Tandem Mass Spectrometry (LC-MS/MS). For determination of a rise in melatonin levels in het evening, only days with 3 valid evening samples will be included in the analyses [23].

The Dim Light Melatonin Onset (DLMO; the time point when melatonin secretion rises over a predefined threshold in the evening [66]) will be calculated after measuring the melatonin evening curve. The DLMO is a reliable estimate of circadian phase position [66].

#### Secondary outcome measures (circadian rhythm)

The circadian rhythm during pregnancy will be studied through actiwatches and the PSQI.

### Actigraphy

Actigraphy, the continuous assessment of activity with a watch-sized non-dominant wrist-worn recorder (Actiwatch Spectrum, Philips Respironics, Pittsburgh, USA), is a validated technique to obtain estimates of sleep and rest-activity rhythms [23, 67, 68]. Sleep analyses software (Actiware 6.0, Philips Respironics, Pittsburgh, USA) will be used to obtain estimates of sleep parameters. The software will calculate end and start of sleep. Further, assumed sleep (the difference between the end and the start of sleep), actual sleep time (amount of sleep determined by algorithm), sleep onset latency (the time between lights out and sleep onset) and sleep efficiency (the percentage of actual sleep time between sleep onset and final awakening, excluding sleep onset latency) will be calculated [23]. Rest-activity rhythms will be calculated, using different actimetric variables, such as interdaily stability and intradaily variability [23].

### PSQI

The PSQI, a structured 19-item self-questionnaire with 5 additional items reported by bedpartner, assesses sleep quality, including a wide variety of factors, such as sleep duration and latency [69]. The sum score ranges from 0 to 21 points, with higher values corresponding to lower sleep quality [69]. These 19 items generate 7 component scores: subjective sleep quality, sleep latency, sleep duration, habitual sleep efficiency, sleep disturbances, use of sleeping medication and daytime dysfunction [69].

A cut-off of 5 will be applied, where a score of <5 indicates poor sleep and ≥5 indicates good sleep [69]. The PSQI has a sensitivity of 89.6 % and a specificity of 86.5 % [69].

The PSQI will be assessed on a monthly base and weekly in the intervention period.

#### Secondary outcome measures (infant)

As infant outcome measures, birth outcomes, infant stress response, infant long-term cortisol exposure and behaviour will be studied.

### Birth outcomes

After birth (T4), information will be obtained regarding complications during delivery and childbirth from the medical records: hypertension, pre-eclampsia, delivery aspects (duration, start with or without induction, augmentation, method of pain relief if any, instrumental delivery, caesarean section, hospital admission) and first week complications after delivery.

### Infant stress response

To measure the infant stress response, we will collect saliva cortisol before and 15, 30 and 45 min after a routine vaccination at T5. We will use the same procedures and analyses as with the mothers.

### Infant long-term cortisol exposure

To measure the long-term cortisol (intra-uterine) exposure, we will collect hair samples of the infant at T5. We will use the same procedures and analyses as with the mothers.

### Infant behaviour

To study infant behaviour, we will assess the questionnaires Mother and Baby Scales (MABS) and Child Behaviour Checklist (CBCL).

### MABS

We ask mothers (and fathers, if available) to fill out the MABS, a questionnaire consisting of various subscales [70]. In this study, we use 3 subscales of the MABS: infant alertness-responsiveness, unsettled-irregular behaviour of the infant and lack of confidence in care-taking [70], resulting in a 36-item questionnaire.

The unsettled-irregular behaviour scale consists of 8 items (e.g. ‘After feeds, I’ve used rocking or cuddling to settle my baby), the alertness-responsiveness scale of 15 items (e.g. ‘My baby watches my face’) and the lack of confidence in feeding of 13 items (e.g. ‘I’ve felt clumsy in caring for my baby’). Parents are asked to score the various statements with a score between 0 (not at all) and 5 (very much/often), which results in a sum score of 0–180. The subscale sum scores range from 0 to 75 for infant unsettled-irregular behaviour, from 0 to 40 for infant alertness-responsiveness and from 0 to 65 for lack of confidence in care-taking [70]. A higher score on the infant unsettled-irregular behaviour scale correlates to more irregular behaviour, whereas a higher score on the infant alertness-responsiveness scale points to more alert behaviour [71]. A higher score on the lack of confidence scale suggests that the mother is less confident in taking care of the baby. Reliability of the MABS ranges from 0.81 to 0.93 (Cronbach’s α), depending on the used subscale [70].

### CBCL

We ask mothers (and fathers, if available) to fill out the CBCL/1.5–5, a diagnostic 99-item questionnaire which quantifies skills and behavioural problems. The questionnaire consists of different scales: emotionally reactive, anxious/depressed, somatic complaints, withdrawn, sleep problems (Internalizing Problems), attention problems and aggressive behaviour (Externalizing Problems) [72]. Together, the Externalizing and Internalizing Problems form the Total Problems [72]. Items can be scored with 0 (not at all), 1 (a little or sometimes) or 2 (clearly or often), resulting in different sum scores for the different scales. Norms for these scales were constructed, using a large representative sample of children [72].

Reliability of the CBCL is 0.85 [73].

#### Chronotype

The participant’s chronotype will be verified at screening, since evening types are found to be more prone to depression than morning types [74, 75]. In this study, this will be assessed with the Munich Chronotype Questionnaire (MCTQ), a structured 19-item self-report measure of an individual’s chronotype, based on sleep times, self-reported light exposure and self-assessed chronotype (extreme, moderate or slight early, normal or slight, moderate or extreme late), taking rest and working days in consideration [76]. The participant can be classified to 1 of the 7 chronotypes by utilizing data on the participant’s midsleep phase and sleep debt [76]. Sum score ranges from 16 to 86, with the lowest score indicating extreme late chronotypes. The MCTQ correlates well with the Horne-Östberg Morningness-Eveningness Questionnaire – especially the MCTQ-assessment of time of midsleep (*r* = −0.73 on free days and *r* = −0.61 on workdays) [77].

### Statistical analysis

#### General

Data will be analysed using SPSS 21.0. For treatment effect analyses, we will apply an intention-to-treat procedure, since none of the participants will switch to another condition and we will include all observations of all participants until study end or withdrawal.

If necessary, skewed continuous variables will be transformed to normality prior to the analyses.

For the continuous variables and categorical variables that are assessed more than twice, we will deploy linear mixed models and generalized linear mixed models respectively. These models use all available data and account for within-subject correlation over time. They can also be used to adequately deal with possible baseline imbalances. Variables measured only once will be compared between the randomized groups using the unpaired t-test, or the Chi-Square test in case of categorical variables. All effect parameters will be supplied with a 95 % confidence interval.

Primary outcome analysis will be first crude, than adjusted. If despite randomization, prognostically important factors differ between the groups, they will be adjusted for supplemental analyses.

#### Primary outcomes

Changes in HAM-D and EPDS rating scale scores over time will be analysed using generalized linear mixed models. Differences from baseline of HAM-D and EPDS scores at every time point will be the dependent variables, time will be the within-subject factor and treatment (BLT versus DRLT) will be the between-subjects factor.

Additional analysis will use alternative definitions of change: instead of the numerical difference of scores before and after, we will use a dichotomous response variable where improvement of ≥ 50 % to a final score ≤ 8 on HAM-D and ≤ 5 on EPDS is defined as success, otherwise not. Finally, we will perform sensitivity analysis to examine robustness of the findings with other methods using data imputation (last observation carried forward multiple imputation).

#### Secondary outcomes

For saliva cortisol, areas under the curves for the morning and evening (i.e. 7 to 9 AM and 8 to 11 PM) will be calculated for subsequent analyses [23]. For saliva melatonin, areas under the curves for the evening (i.e. 8 to 11 PM) will be calculated for subsequent analyses [23].

Continuous outcomes, e.g. saliva cortisol and melatonin, will be tested with the unpaired t-test and linear mixed model. Categorical outcomes, e.g. sleep quality, will be tested with Chi-Square Test and generalized linear mixed model.

#### Covariates

Potential covariates for the mother are psychiatric history, ethnicity, level of education, parity, gestational age (duration of pregnancy at study entry), substance use, chronotypes, duration of actual depression and other psychiatric or psychotherapeutic treatment interventions. These factors might affect depressive symptoms during pregnancy. After pregnancy, we will additionally correct for complications during delivery, breastfeeding and objective and subjective sleep parameters, since these factors might influence depressive symptoms after delivery.

Potential covariates for the infant are head circumference, congenital malformations, Apgar-score and neonatal admission at Neonatal Intensive Care Unit.

## Discussion

We have presented a protocol for an RCT of light therapy for antepartum depression. Two earlier conducted RCT’s have shown the effects of BLT in pregnant women with depression, but studied only a small sample size (*n* = 10 and *n* = 27) [41, 42]. In this trial, we will study the effects of BLT on antepartum depression in a larger sample (*n* = 150) and in addition to the earlier conducted studies, we will not only study the effects of BLT on the mother, but on the infant as well. Moreover, we will study the effects of BLT on maternal endocrine levels and maternal circadian rhythm.

BLT will benefit pregnant women, for they will receive immediate treatment for their depressive symptoms. Psychotherapists are not always available, which would postpone treatment. Also, antidepressant medication is not immediately effective. However, BLT may be effective in as little as 1 week [55]. Moreover, the adverse effects of BLT are mild and short-lived.

The unborn child would benefit from this alternative treatment, for BLT does not cause adverse effects in the unborn child. Also, since BLT may be effective in a short time period, the risks associated with maternal depression (such as lower birth weight) may be diminished.

BLT would benefit society, since BLT has lower costs than treatment with antidepressant medication or psychotherapy.

Finally, since this RCT would be the first to study the effects of BLT in the infant, it would contribute to the understanding of the role of BLT, depression and the maternal HPA-axis in the developing foetus.

Thus, if BLT reduces depressive symptoms in pregnant women, it will provide an alternative, non-pharmalogical treatment for psychotherapy and antidepressant medication in treating antepartum depression. BLT combines direct availability, sufficient efficacy, low costs and high safety, taking the safety for the unborn child into account as well. Moreover, it does not require good language skills and it can be administered at home. These considerations make BLT for treating depression in pregnant women relevant, especially in urban multi-ethnic populations with high prevalence of depression and a low level of personal resources.

## Abbreviations

ACTH: Adrenocorticotrophic hormone
BLT: Bright light therapy
CRF: Adrenocorticotrophic hormone-releasing factor
DLMO: Dim light melatonin onset
DRLT: Dim red light therapy
DSM: Diagnostic and Statistical Manual of mental disorders
EPDS: Edinburgh Postnatal Depression Scale
GP: General practitioner
GR: Glucocorticoid receptor
HAM-D: Hamilton rating scale for depression
HPA: Hypothalamus-pituitary-adrenal gland
MABS: Mother And Baby Scales
MCTQ: Munich Chronotype Questionnaire
PSQI: Pittsburgh Sleep Quality Index
PVN: Paraventricular nucleus
SCID: Structured Clinical Interview for DSM disorders
SIGH-SAD: Structured Interview Guide for the Hamilton depression rating scale - Seasonal Affective Disorder version
